# Hepcidin deficiency impairs hippocampal neurogenesis and mediates brain atrophy and memory decline in mice

**DOI:** 10.1186/s12974-023-03008-0

**Published:** 2024-01-09

**Authors:** Xue Bai, Bing Wang, Yiduo Cui, Siqi Tian, Yi Zhang, Linhao You, Yan-Zhong Chang, Guofen Gao

**Affiliations:** Ministry of Education Key Laboratory of Molecular and Cellular BiologyHebei Key Laboratory of Animal Physiology, Biochemistry and Molecular Biology, Hebei Research Center of the Basic Discipline of Cell Biology, College of Life Sciences, Hebei Normal University, Shijiazhuang, 050024 Hebei China

**Keywords:** Hepcidin, Iron, Alzheimer’s disease, Hippocampal neurogenesis, Cognitive dysfunction

## Abstract

**Background:**

Hepcidin is the master regulator of iron homeostasis. Hepcidin downregulation has been demonstrated in the brains of Alzheimer’s disease (AD) patients. However, the mechanism underlying the role of hepcidin downregulation in cognitive impairment has not been elucidated.

**Methods:**

In the present study, we generated *GFAP*-Cre-mediated hepcidin conditional knockout mice (*Hamp*^*GFAP*^ cKO) to explore the effect of hepcidin deficiency on hippocampal structure and neurocognition.

**Results:**

We found that the *Hamp*^*GFAP*^ cKO mice developed AD-like brain atrophy and memory deficits. In particular, the weight of the hippocampus and the number of granule neurons in the dentate gyrus were significantly reduced. Further investigation demonstrated that the morphological change in the hippocampus of *Hamp*^*GFAP*^ cKO mice was attributed to impaired neurogenesis caused by decreased proliferation of neural stem cells. Regarding the molecular mechanism, increased iron content after depletion of hepcidin followed by an elevated level of the inflammatory factor tumor necrosis factor-α accounted for the impairment of hippocampal neurogenesis in *Hamp*^*GFAP*^ cKO mice. These observations were further verified in *GFAP* promoter-driven hepcidin knockdown mice and in *Nestin*-Cre-mediated hepcidin conditional knockout mice.

**Conclusions:**

The present findings demonstrated a critical role for hepcidin in hippocampal neurogenesis and validated the importance of iron and associated inflammatory cytokines as key modulators of neurodevelopment, providing insights into the potential pathogenesis of cognitive dysfunction and related treatments.

**Graphical Abstract:**

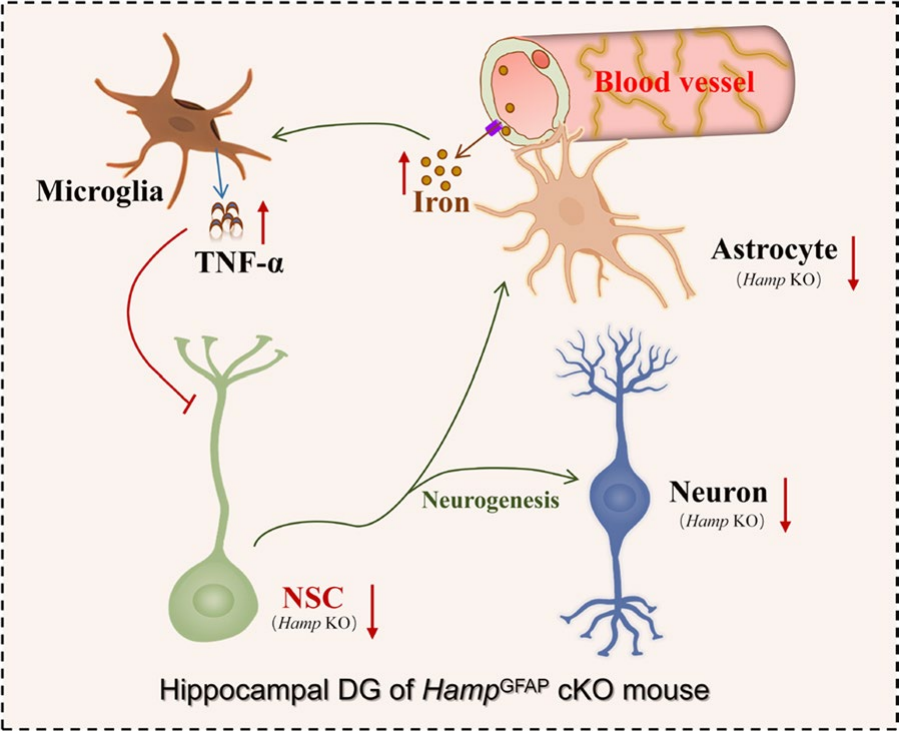

**Supplementary Information:**

The online version contains supplementary material available at 10.1186/s12974-023-03008-0.

## Background

Hepcidin is the major peptide hormone that regulates systemic iron homeostasis; it modulates the activity of the only known cellular iron exporter ferroportin 1 (FPN1), thereby reducing iron release from target cells [1, 2]. In the periphery, hepcidin is mainly produced by hepatocytes and secreted into the plasma, inhibiting iron absorption in the intestines and iron release from macrophages and thus decreasing the plasma iron concentration [3]. In the central nervous system, hepcidin is expressed in neurons and glia in various brain regions [4, 5]. The aberrant expression of hepcidin and the dysregulation of iron metabolism have been reported to be associated with the pathogenesis of neurodegenerative diseases, such as Alzheimer’s disease (AD) [6, 7].

Studies have shown that hepcidin expression is significantly reduced in multiple brain regions, including the hippocampus, entorhinal cortex, and superior frontal gyrus, in AD patients and animal models, correlated with the excessive iron deposition observed in these regions [7]. Upregulation of hepcidin in the brain significantly attenuates brain iron accumulation in iron-overloaded rats and APP/PS1 mice [8, 9]. Additionally, hepcidin protects neurons from hemin-mediated injury [10]. The expression level of hepcidin is age-dependent and is closely associated with inflammatory responses in the nervous system [11–13]. Iron dysregulation is a key pathogenic factor in neurodegenerative diseases [14–16]. However, the roles of hepcidin under physiological and pathological conditions have not been fully elucidated.

Our previous studies have shown that global hepcidin knockout (*Hamp*^−/−^) mice exhibit gradually increasing iron levels with age in the cerebral cortex and hippocampus [17]. We demonstrate that hepcidin secreted by astrocytes plays a critical role in regulating brain iron uptake at the blood‒brain barrier (BBB) by controlling FPN1 internalization and degradation in microvascular endothelial cells. Consistently, hepcidin overexpression in astrocytes improves the cognitive function of AD model mice by reducing neuronal iron levels in the cortex and hippocampus, as well as suppressing oxidative damage and inflammation [9, 18]. However, the mechanisms underlying the role of hepcidin deficiency in the pathogenesis of early-stage cognitive dysfunction, particularly in association with impaired hippocampal structure and function, remain unclear.

In the present study, we generated *GFAP*-Cre-mediated *Hamp* conditional knockout (*Hamp*^*GFAP*^ cKO) mice and used *Hamp*^flox/flox^ mice as controls to explore the effect of hepcidin deficiency on hippocampal development and neurocognition. GFAP is expressed not only in mature astrocytes, but also in neural stem/progenitor cells (NSCs) located in the subgranular zone (SGZ) of the hippocampal dentate gyrus (DG) [19]. Consequently, *GFAP*-Cre-mediated hepcidin depletion was observed in both astrocytes and granule cells of the hippocampal DG. Our findings showed that hepcidin deficiency in *Hamp*^*GFAP*^ cKO mice impaired hippocampal neurogenesis and mediated cognitive dysfunction. The present study provides new insight into the potential mechanisms of hepcidin deficiency-related neurodegeneration and cognitive decline and highlights new targets for the treatment of cognitive disorders.

## Methods

### Animals

*Hamp*^flox/+^ mice, *GFAP*-Cre, and *Nestin*-Cre mice were purchased from Cyagen Biosciences Co., Ltd. (Suzhou, China), and their offspring were genotyped to obtain *Hamp*^GFAP^ cKO (*Hamp*^flox/flox^; *GFAP*-Cre) and *Hamp*^Nes^ cKO (*Hamp*^flox/flox^; *Nestin*-Cre) mice. The primers used for genotyping are listed in Additional file 1: Table S1. The littermate transgenic mice were assigned to experimental groups according to their genotypes. For the groups with more than the required number of animals, random selection was conducted within the group by lottery method. Wild-type (WT) C57BL/6 mice were purchased from Beijing Experimental Animal Research Center. After one week of acclimatization, animals were randomly divided into experimental groups.

Mice were housed in dedicated mouse cages in the SPF animal chambers and maintained at 22 ± 1 ℃, 40–55% humidity, and a 12 h/12 h light–dark cycle. All procedures were carried out in accordance with the National Institutes of Health Guide for the Care and Use of Laboratory Animals, and were approved by the Animal Ethics Committee of Hebei Normal University (authorization number: 2020LLSC21).

### Injections and treatments

To detect proliferating cells in the adult hippocampus, 3-month-old *Hamp*^*GFAP*^ cKO and control mice were intraperitoneally injected with BrdU at a dose of 180 mg/kg and killed after 12 h to assess the number of BrdU-labeled cells in the hippocampal SGZ area in different mice.

To achieve hippocampal iron overload, 6-week-old C57BL/6 male mice were injected with 1 µL ferric ammonium citrate (FAC; 0.5 µg/µL) or saline into each side of the hippocampus: − 2 mm anteroposterior and ± 1.7 mm lateral to bregma and − 1.6 mm ventral from the dura, as reported previously [20]. The mice were killed after 2 weeks for analysis.

To achieve hepcidin knockdown in the hippocampus, 8-week-old C57BL/6 male mice were intrahippocampally injected with 1 µL pAAV-*GFAP*:sh*Hamp* or pAAV-*GFAP*:shScramble control [21] at a concentration of 2 µg/µL. Following the injection, the mouse brains were electroporated using square wave pulses with an Electro Square Porator ECM830 (Harvard Apparatus BTX Inc., USA), as described previously [20]. The mice were killed for analysis 3 weeks after the injection.

### Morris water maze (MWM) test

Behavior analyses were performed blindly. Briefly, mice of the different groups were assigned numbers in a random manner. The experimenters were blinded to the animal genotypes during the test. After exporting the data from the automatic video-tracking system, the genotype of each mouse was revealed.

The MWM test was performed using an MWM Video Analysis System V2.0 (Anhui Zhenghua Biologic Apparatus Facilities Co., Anhui, China) as previously described [17]. First, the mice were trained for two days to find a visible escape platform from different quadrants. Then, the platform was hidden 1 cm under the water. In a 90-s trial, the time spent swimming and distance travelled by each mouse before it reached the hidden platform were recorded for four consecutive days. The platform was removed on the fifth day. The number of times that the mouse crossed the former location of the platform in 90 s and the percentage of time spent in the target quadrant were recorded for analysis.

### Elevated plus maze test

The elevated plus maze consisted of a pair of open arms and a pair of closed arms linked with a central platform, and it was elevated 56 cm above the ground. Each mouse was placed into the center of the platform, and its activity was recorded for 5 min by a video-tracking system (SMART v3.0, RWD Life Science Co., Ltd, Shenzhen, China). The time spent and distance travelled in the closed arms, open arms, and center zone were analyzed. Before the next mouse was tested, the entire apparatus was wiped with alcohol.

### Open field test

The open field apparatus consisted of a square box that was divided into center and peripheral zones. The mice were placed in the central zone, and their locomotor activity over 10 min was recorded. The percentage of time spent in and distance travelled in the center zone and the number of entries into the center zone were analyzed by an automatic video-tracking system (SMART v3.0, RWD Life Science Co., Ltd, Shenzhen, China). Before the next animal was tested, the apparatus was wiped thoroughly with alcohol.

### Immunofluorescence and immunohistochemical staining

Dissected brain samples were fixed with 4% paraformaldehyde at 4 °C and then dehydrated with 30% sucrose solution. Sections were cut at 20 μm thickness on a freezing microtome and stored at − 80 °C until use. The frozen sections were restored to room temperature, washed with 0.01 M PBS, and then incubated for 20 min in 3% H_2_O_2_ to block endogenous peroxidase activity. For antigen retrieval, the sections were microwaved in 0.01 M sodium citrate for 15 min, and then washed with PBS after cooling to room temperature. For BrdU staining, the sections were treated with 1 M HCl for 30 min on ice, 2 M HCl for 10 min at room temperature and 20 min at 37 °C, and then neutralized with 0.1 M borate buffer for 10 min at room temperature. The samples were blocked with 10% goat serum for 50 min at 37 °C. After washing, primary antibodies were added and incubated overnight at 4 °C. The primary antibodies included mouse anti-NeuN (ab104224, Abcam, USA), rabbit anti-Pax6 (PRB-278P, Covance, USA), rabbit anti-Ki67 (ab15580, Abcam), rabbit anti-doublecortin (DCX; #4604, CST, USA), rat anti-BrdU (ab6326, Abcam), rabbit anti-ferritin light chain (FtL; ab109373, Abcam), rabbit anti-Iba1 (#17198, CST), and rabbit anti-cleaved caspase-3 (#9661, CST) antibodies.

For immunohistochemical staining, after washing, the sections were incubated with corresponding biotinylated secondary antibodies for 1 h at 37 °C. After washing, the sections were treated with avidin-biotinylated horseradish peroxidase complex for 50 min at 37 °C and then stained with a DAB kit. The staining was stopped by rinsing with tap water. The slices were dehydrated in graded alcohol solutions, cleared with xylene and sealed with resin adhesive. Finally, the sections were photographed with a Zeiss LSM710 microscope. For immunofluorescence staining, Alexa Fluor 488- or 594-conjugated secondary antibodies were added and incubated at 37 °C for 1 h. Nuclei were then counterstained with DAPI for 4 min. After washing and mounting, the slices were photographed by a TissueFAXS Fluorescence System or by a fluorescence confocal microscope (Olympus FV3000, Japan). For each analysis, brain slices of similar stereotaxic coordinates from more than three mice per group were stained, and the cell number or fluorescence intensity was quantified.

### Nissl staining

Frozen sections were restored to room temperature, washed with distilled water, and then incubated in Nissl Stain Solution at 55 °C for 50 min. After washing with distilled water, the sections were rapidly differentiated with 95% ethanol, dehydrated with 100% ethanol, cleared with xylene and mounted with Permount Mounting Medium. Brain slices of similar stereotaxic coordinates from more than three mice per group were used for staining and photographed for analysis.

### Measurement of apoptosis

Apoptosis in the hippocampus was detected by a terminal deoxynucleotidyl transferase-mediated dUTP nick-end labeling (TUNEL) staining kit following the manufacturer’s protocol (TUNEL BrightGreen Apoptosis Detection Kit, Vazyme, Nanjing, China). Nuclei were counterstained with DAPI. After mounting, the slices were photographed by a fluorescence confocal microscope.

### Western blot

Tissue of hippocampus was homogenized and sonicated in RIPA buffer containing 1% NP40 and protease inhibitor cocktail tablet (Roche, Germany). After centrifugation at 12,000*g* for 20 min at 4 °C, the supernatant was collected and protein concentration was determined using a BCA protein assay kit. Approximately 30 μg of total protein for each sample was resolved by SDS-PAGE with 10% or 12% gels, and then transferred onto NC membranes. The blots were blocked in TBS-T buffer (20 mM Tris–HCl, 137 mM NaCl and 0.1% Tween-20, pH 7.6) containing 5% non-fat milk for 1.5 to 2 h at room temperature, followed by incubation with the specific primary antibody overnight at 4 °C. The primary antibodies included mouse anti-β-actin (CW0096, Cwbio, Beijing, China), anti-GAPDH (60,004–1-Ig, Proteintech, Wuhan, China), rabbit anti-FtL (ab109373, Abcam), rabbit anti-glutathione peroxidase 4 (GPx4; ab125066, Abcam), rabbit anti-acyl-CoA synthetase long-chain family member 4 (ACSL4; ab155282, Abcam), rabbit anti-nerve growth factor (NGF; DF6061, Affinity Biosciences, Changzhou, China), and rabbit anti-brain-derived neurotrophic factor (BDNF; ab108319, Abcam) antibodies. After washing with TBS-T, the membranes were then incubated with HRP-conjugated secondary antibody for 1.5 h at room temperature, detected using an enhanced chemiluminescence detection kit (Amersham, UK) and visualized by a chemiluminescence imager.

### Quantitative reverse-transcription PCR (qRT-PCR)

Total RNA in the mouse hippocampus was extracted using TRIzol reagent (Invitrogen, USA). The cDNA was then reverse-transcribed from 1 μg of the extracted RNA using M-MLV reverse transcriptase (TaKaRa, Japan) and Oligo-dT primers. The PCR amplification was performed with SYBR green PCR Master Mix (Cwbio, China), with specific primers of target genes listed in Additional file 1: Table S2. The expression level of GAPDH was used as internal reference. The equation 2^−ΔΔCt^ was used to calculate the relative mRNA expression level of the target genes.

### Perl’s staining

Brain sections were incubated with 3% H_2_O_2_ for 20 min at room temperature and then stained with Perl’s stain solution (2% potassium ferrocyanide and 2% hydrochloric acid) at 37 °C for 14 h. The staining was enhanced by incubation in DAB for 5 min, and the reaction was terminated by tap water. The sections were dehydrated in graded alcohol solutions, cleared with xylene, and sealed with neutral gum. The sections were photographed with a Zeiss LSM710 microscope.

### Inductively coupled plasma-mass spectrometry (ICP-MS) assay

The total iron content in the hippocampus was measured by ICP-MS. The samples were dried and resuspended in 500 μL of 65% nitric acid. After heating at 90 °C for 20 min, 500 μL 30% H_2_O_2_ was added and incubated for 15 min at 70 °C followed by a 6 h incubation at 100 °C. The digested samples were then dissolved in 2 mL of ultrapure water and subjected to ICP-MS. Iron concentrations were calculated according to a standard curve and then normalized to the dry tissue weight.

### Statistical analysis

The data were presented as the mean ± SEM. All data were checked for normality distribution by using the Shapiro–Wilk test, and those passed the normality tests were processed for statistical analysis by the two-tailed Student’s *t*-test. The *p* values of < 0.05 were considered as statistically significant differences. Details of the number of independent experiments are provided in the figure legends.

## Results

### *GFAP*-Cre-mediated hepcidin knockout induced cognitive impairment and anxiety-like behaviors

**Fig. 1:**
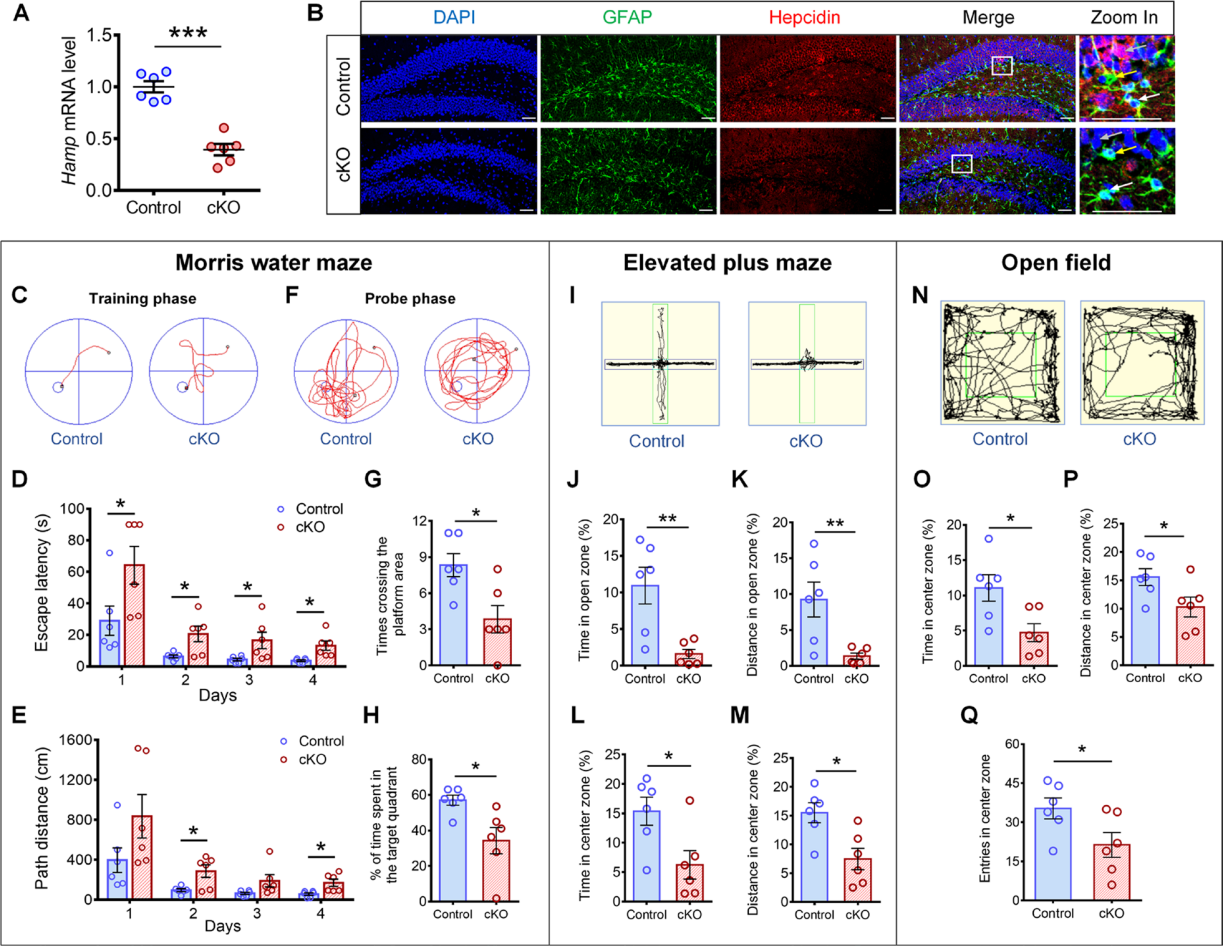
*GFAP*-Cre mediated hepcidin depletion induced cognitive impairment and anxiety-like behavior. **A** qRT-PCR to detect *Hamp* mRNA levels in 3-month-old *Hamp*^*GFAP*^ cKO mice and *Hamp*^*flox/flox*^ control mice. **B** Immunofluorescence staining of hepcidin and GFAP expression in the DG of cKO and control mice. White arrows point out astrocytes; yellow arrows point out GFAP-positive NSCs; gray arrows point out granule neurons. Scale bar: 50 µm. **C**–**H** The spatial learning and memory of 3-month-old *Hamp*^*GFAP*^ cKO and control mice were assessed in MWM test. Representative mouse travel tracks at day 4 (**C**) and the quantifications of escape latency (**D**) and distance (**E**) over 4 days are shown. Representative mouse travel tracks in the probe test at day 5 (**F**), the number of times crossing the platform location (**G**) and percentage of time spent in the target quadrant (**H**) were analyzed. **I**–**M** In elevated plus maze, the representative mouse travel tracks (**I**), percentages of time (**J**) and distance (**K**) spent in the open zone, and percentages of time (**L**) and distance (**M**) in the center area were analyzed in 6-month-old *Hamp*^*GFAP*^ cKO and control mice. **N**–**Q** In open field test, the representative mouse travel tracks (**N**), percentages of time (**O**) and distance (**P**) spent in the center zone, and entries into the center zone (**Q**) were analyzed in 6-month-old *Hamp*^*GFAP*^ cKO and control mice. Data were expressed as mean ± SEM, *n* = 6 per group. *p < 0.05, **p < 0.01 and ***p < 0.001

*Hamp*^*GFAP*^ cKO mice and the control *Hamp*^flox/flox^ mice were genotyped according to the presence of flox- and *GFAP*-Cre-specific bands (Additional file 1: Fig. S1A). The decrease in *Hamp* mRNA levels in the hippocampus of *Hamp*^*GFAP*^ cKO mice was confirmed by qRT-PCR (Fig. 1A). In addition, immunofluorescence showed that hepcidin was depleted not only in astrocytes, but also in GFAP-positive NSCs in the hippocampal SGZ of *Hamp*^*GFAP*^ cKO mice (Fig. 1B), as well as in dentate granule cells derived from GFAP-positive NSCs (Fig. 1B).

The hippocampus plays a crucial role in memory and anxiety-like behavior. Thus, the performance of 3- and 6-month-old *Hamp*^*GFAP*^ cKO mice and the control *Hamp*^*flox/flox*^ mice in the MWM, elevated plus maze, and open field tests was assessed. In the MWM test, 3-month-old cKO mice had a significantly longer escape latency and travelled a longer distance to reach the platform than control mice during the training stage (Fig. 1C–E). After removing the platform, the cKO mice spent less time in the target quadrant and crossed the platform location fewer times (Fig. 1F–H). Similar changes were also observed in 6-month-old cKO mice compared to control mice (Additional file 1: Fig. S1B–E). In the elevated plus maze test, 3-month-old cKO mice spent a smaller percentage of time and travelled a shorter distance in the open arms (Additional file 1: Fig. S1F–I), whereas 6-month-old cKO mice spent a substantially lower percentage of time and travelled a markedly shorter distance in both the open arms and the central area (Fig. 1I–M) than the control mice. Consistently, in the open field test, 6-month-old cKO mice showed a significantly smaller percentage of time, a shorter distance and fewer entries in the central zone (Fig. 1N–Q). These results indicate that hepcidin knockout in GFAP-positive cells and dentate granule neurons derived from GFAP-positive NSCs induces cognitive impairment and anxiety-like behavior in mice.

### *Hamp*^*GFAP*^ cKO mice had hippocampal atrophy and a dramatic reduction in the number of neurons in the DG

**Fig. 2:**
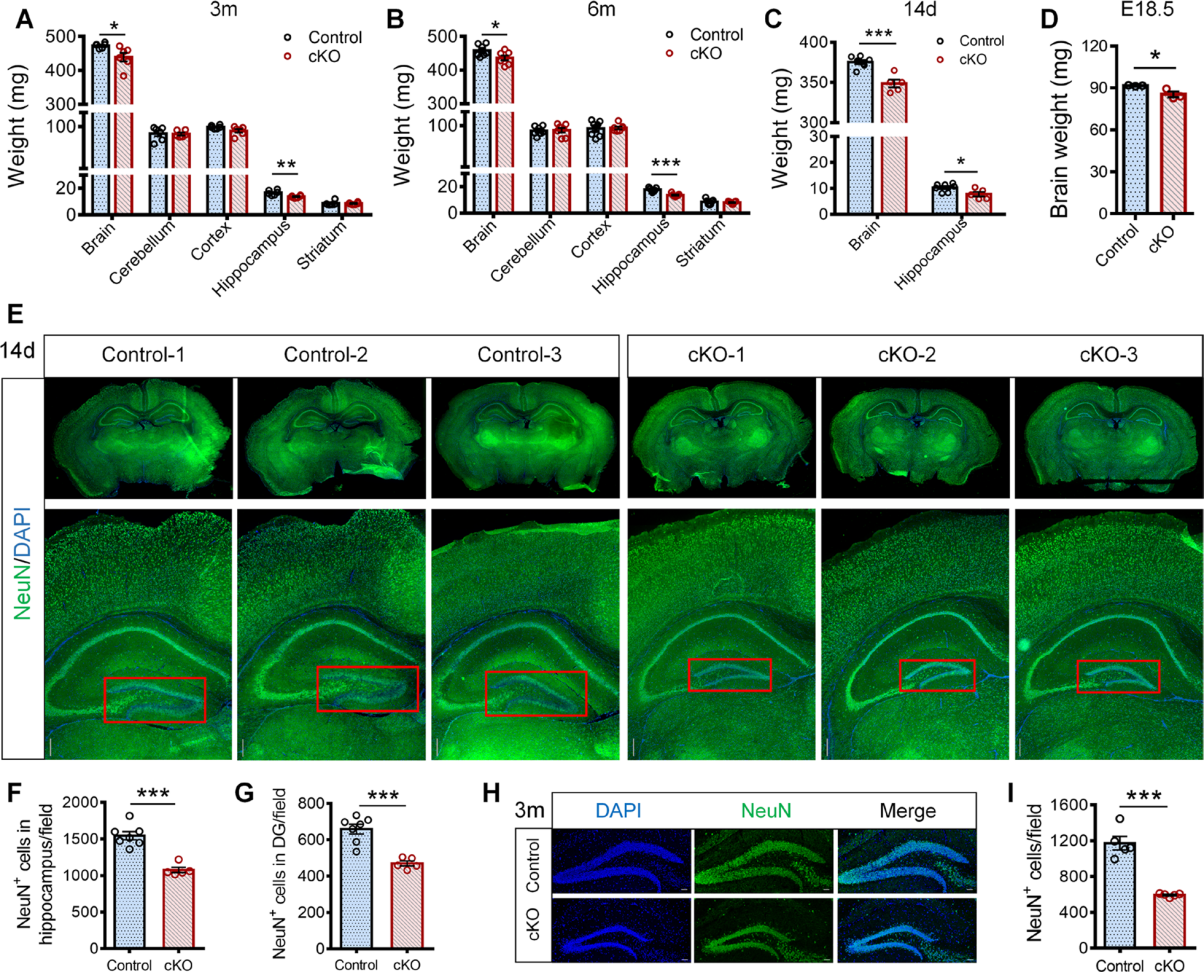
*Hamp*^*GFAP*^ cKO mice had hippocampal atrophy and decreased number of neurons in DG. **A**, **B** The weights of total brain, cerebellum, unilateral cortex, hippocampus and striatum were measured in 3-month-old (**A**) and 6-month-old (**B**) *Hamp*^*GFAP*^ cKO mice with their respective control mice (*n* = 6 and *n* = 7 per group in 3- and 6-month-old mice, respectively). **C** The weights of brain and hippocampus in 14-day-old *Hamp*^*GFAP*^ cKO and control mice (*n* = 7 and 5, respectively). **D** The brain weight of the E18.5-day-old *Hamp*^*GFAP*^ cKO and control mice (*n* = 3 per group). **E** Representative images of immunofluorescence staining of NeuN^+^ cells in 14-day-old *Hamp*^*GFAP*^ cKO and control mice. Scale bar: 200 µm. **F**, **G** Quantifications of the number of NeuN^+^ cells in hippocampus (**F**) and in DG region (**G**) of the *Hamp*^*GFAP*^ cKO and control mice (*n* = 7 and 5, respectively). **H**, **I** Immunofluorescence staining of NeuN^+^ cells (**H**) and the quantification (**I**) in hippocampal DG of 3-month-old *Hamp*^*GFAP*^ cKO and control mice (scale bar: 50 μm, *n* = 5 per group). Data were expressed as mean ± SEM. *p < 0.05, **p < 0.01 and ***p < 0.001

After the mice were killed, the weights of the total brain, cerebellum, unilateral cortex, hippocampus, and striatum were measured. The 3- and 6-month-old *Hamp*^*GFAP*^ cKO mice had significantly lower brain weights and hippocampal weights than the age-matched control mice (Fig. 2A, B). However, there were no significant differences in the weight of the cerebellum, cortex or striatum (Fig. 2A, B) or body weight (Additional file 1: Fig. S2A, B) between cKO mice and control mice. These findings indicate that hippocampal development in *Hamp*^*GFAP*^ cKO mice might be severely affected by hepcidin deficiency. Consistent with this speculation, we found that the weights of the brain and hippocampus in 14-day-old cKO mice were significantly decreased (Fig. 2C) while the body weight of these mice did not change significantly (Additional file 1: Fig. S2C), compared to that of the control mice. Furthermore, changes in brain weight were also found in embryonic day 18.5 fetal cKO mice (Fig. 2D). These findings suggest that the pathological changes in *Hamp*^*GFAP*^ cKO mice may begin in the embryonic stage and persist through the postnatal period.

We assessed hippocampal morphology in *Hamp*^*GFAP*^ cKO and control mice by immunofluorescence staining and Nissl staining. As shown in Fig. 2E, in 14-day-old cKO mice, a strikingly smaller hippocampal DG was observed compared to the control mice. Quantitative analyses showed that the numbers of NeuN^+^ neuronal cells in the hippocampus and DG of cKO mice were significantly reduced (Fig. 2F, G). Furthermore, 3-month-old cKO mice showed a markedly reduced number of NeuN^+^ cells in the DG (Fig. 2H, I; Additional file 1: Fig. S2D, E). These findings demonstrate that *GFAP*-Cre-mediated hepcidin depletion causes hippocampal atrophy in mice, which is likely associated with abnormal neurodevelopment primarily of the hippocampal DG.

### *Hamp*^*GFAP*^ cKO mice exhibited reduced proliferation of hippocampal NSCs and impaired hippocampal neurogenesis

**Fig. 3:**
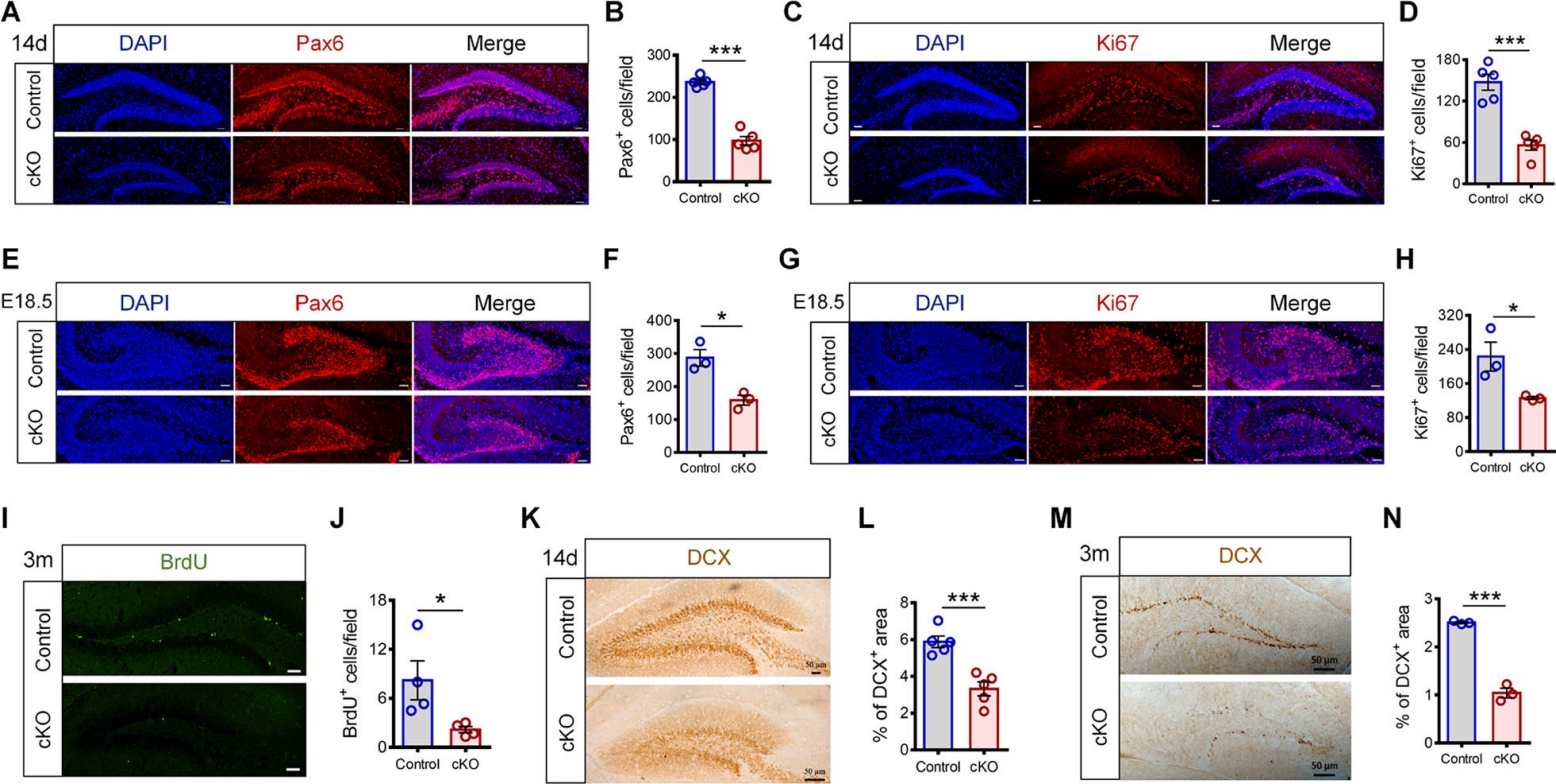
*Hamp*^*GFAP*^ cKO mice exhibited reduced proliferation levels of hippocampal NSCs. **A**–**D** Immunofluorescence staining images and quantifications of Pax6^+^ cells (**A**, **B**) and Ki67^+^ cells (**C**, **D**) in the DG region of 14-day-old *Hamp*^*GFAP*^ cKO and control mice (scale bar: 50 μm, *n* = 5 per group). **E**–**H** Immunofluorescence staining images and quantifications of Pax6^+^ cells (**E**, **F**) and Ki67^+^ cells (**G**, **H**) in the DG region of E18.5-day *Hamp*^*GFAP*^ cKO and control mice (scale bar: 50 μm, *n* = 3 per group). **I**, **J** Immunofluorescence staining images of BrdU^+^ cells (**I**) and quantification (**J**) in the hippocampal DG of 3-month-old *Hamp*^*GFAP*^ cKO and control mice (scale bar: 50 μm, *n* = 4 per group). **K**–**N** Immunohistochemical staining images of DCX^+^ cells and quantification in the hippocampal DG of 14-day-old *Hamp*^*GFAP*^ cKO and control mice (**K** and **L**, *n* = 5 per group) and 3-month-old *Hamp*^*GFAP*^ cKO and control mice (**M** and **N**, *n* = 3 per group). Data were expressed as mean ± SEM. *p < 0.05, and ***p < 0.001

The hippocampal DG is one of the most important brain regions for neurogenesis during the embryonic and postnatal periods [22]. Therefore, we evaluated the effects of hepcidin cKO on the proliferation of hippocampal NSCs. We performed staining for the stem cell marker Pax6 and proliferation marker Ki67. As shown in Fig. 3A–D, the numbers of Pax6^+^ cells and Ki67^+^ cells in the hippocampal DG of 14-day-old *Hamp*^*GFAP*^ cKO mice were significantly lower than those in the control mice. In addition, the numbers of Pax6^+^ cells and Ki67^+^ cells in the hippocampus of embryonic 18.5-day-old cKO mice were also significantly decreased (Fig. 3E–H). Moreover, the changes in the proliferation of adult hippocampal NSCs from 3-month-old cKO and control mice were examined using BrdU pulse labeling. The results showed that the number of BrdU^+^ cells in the DG of cKO mice also decreased significantly (Fig. 3I, J). These results suggest that *GFAP*-Cre-mediated hepcidin depletion leads to impaired proliferation of hippocampal NSCs from the embryonic stage to the postnatal period.

To assess whether neurogenesis in the hippocampus of *Hamp*^*GFAP*^ cKO mice was affected, DCX immunostaining was used to evaluate the changes in newborn neurons. The results showed that the number of DCX^+^ cells in the DG of 14-day-old cKO mice was significantly decreased (Fig. 3K, L), and the DCX intensity in the DG of 3-month-old cKO mice was also substantially decreased (Fig. 3M, N). These findings suggest that *GFAP*-Cre-mediated hepcidin depletion impairs hippocampal neurogenesis.

### Iron content in the hippocampus of *Hamp*^*GFAP*^ cKO mice increased

**Fig. 4:**
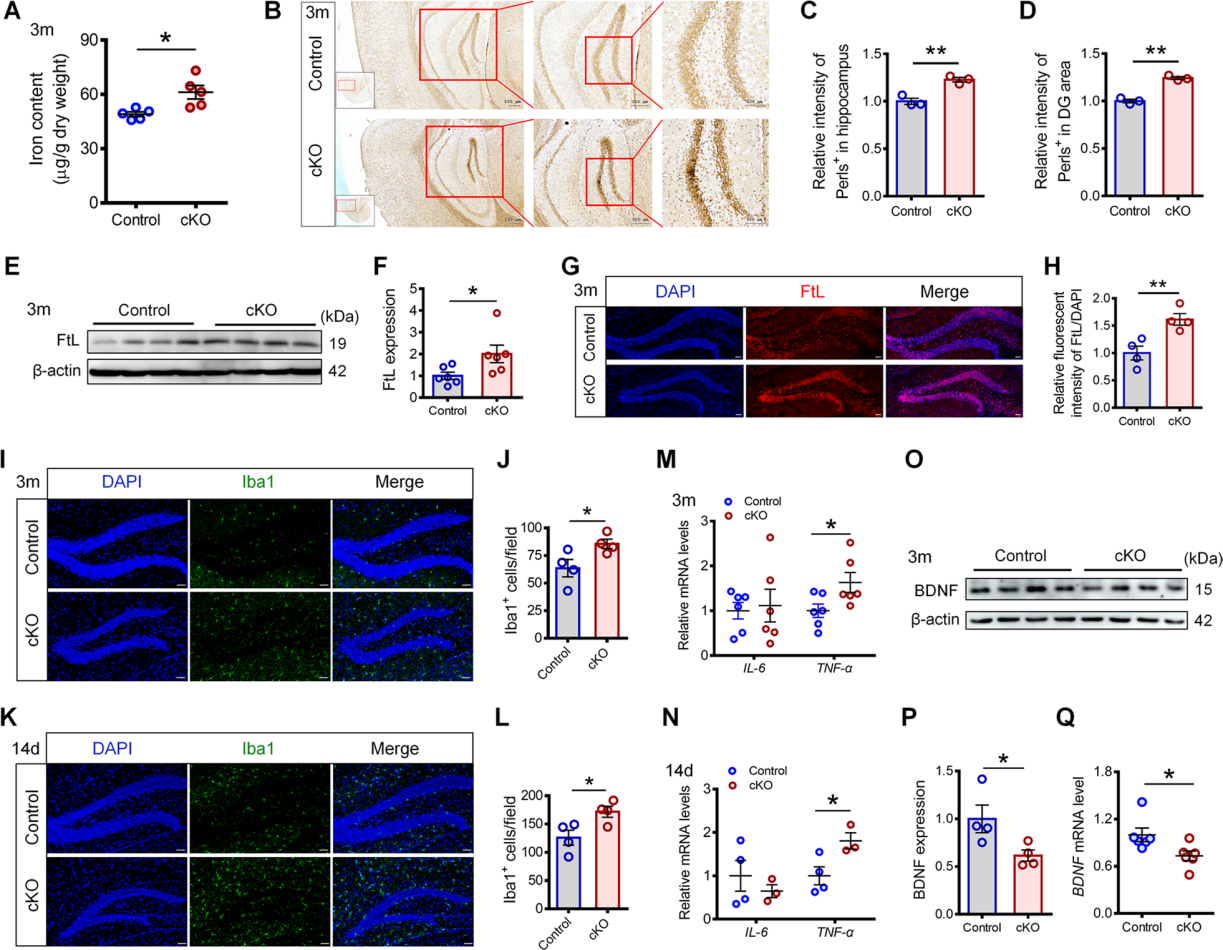
Increased iron content, microglia and TNF-α levels, but decreased BDNF expression were observed in the hippocampus of *Hamp*^*GFAP*^ cKO mice. **A** Iron contents in the hippocampus of 3-month-old *Hamp*^*GFAP*^ cKO and control mice were detected by ICP-MS (*n* = 5). **B**–**D** Iron distribution in the brains of 3-month-old *Hamp*^*GFAP*^ cKO and control mice were observed by Perl’s staining, and relative positive signals in the hippocampus (**C**) and DG region (**D**) were analyzed (*n* = 3 per group). **E–F** Representative blot image (**E**) and quantification (**F**) of the hippocampal FtL expression in 3-month-old *Hamp*^*GFAP*^ cKO and control mice (*n* = 6 per group; β-actin as the internal reference). **G**, **H** Immunofluorescence staining images (**G**) and quantification (**H**) of relative FtL^+^ fluorescence intensity in the hippocampal DG of 3-month-old *Hamp*^*GFAP*^ cKO and control mice (scale bar: 50 μm, *n* = 4 per group). **I**–**L** Immunofluorescence staining images of Iba1^+^ cells and quantifications in the hippocampal DG of 3-month-old (**I**, **J**) and 14-day-old (**K**, **L**) *Hamp*^*GFAP*^ cKO and control mice (scale bar: 50 μm, *n* = 4 per group). **M**, **N** qRT-PCR quantification for detecting the mRNA levels of *TNF-α* and *IL-6* in hippocampus of 3-month-old (**M**) and 14-day-old (**N**) *Hamp*^*GFAP*^ cKO and control mice (*n* = 6 per group in M; *n* = 4 and 3 in N). **O**–**Q** Representative blot images (**O**) and quantification (**P**) of BDNF protein levels in the hippocampus of 3-month-old *Hamp*^*GFAP*^ cKO and control mice (*n* = 4 per group; β-actin as the internal reference), and qRT-PCR quantification of *BDNF* mRNA levels (**Q**, *n* = 6 per group). Data were expressed as mean ± SEM. *p < 0.05, and **p < 0.01

To explore the role of hepcidin in regulating iron homeostasis, we examined hippocampal iron levels in 3-month-old *Hamp*^*GFAP*^ cKO mice and control mice. The results of ICP-MS showed that the iron content in the hippocampus of cKO mice was significantly higher than that of control mice (Fig. 4A). Perl’s iron staining showed an increased iron signal intensity in the hippocampus and the DG of cKO mice (Fig. 4B–D). These observations were consistent with the increased brain iron content in the *Hamp*^−/−^ mice previously reported [17]. Moreover, the expression level of the iron storage protein FtL was also increased in the hippocampus of cKO mice (Fig. 4E, F), which reflected an increase in iron content [23]. Consistently, the immunostaining results also showed elevated FtL expression in the hippocampal DG of cKO mice (Fig. 4G, H). These results indicate that *Hamp*^*GFAP*^ cKO mice have an increased iron content in the hippocampus.

Cellular iron overload is often associated with enhanced apoptosis [24–26]. Therefore, we assessed whether the number of apoptotic cells increased in the hippocampus of *Hamp*^*GFAP*^ cKO mice and whether apoptosis accounted for hippocampal atrophy. Based on TUNEL and immunofluorescence staining, we did not observe a significant increase in the number of apoptotic cells (Additional file 1: Fig. S3A) or in cleaved caspase-3 expression (Additional file 1: Fig. S3B) in the hippocampus of cKO mice compared to control mice. Furthermore, we measured the expression levels of ferroptosis-related proteins GPx4 and ACSL4 [27, 28]. No significant changes in the levels of GPx4 or ACSL4 in the hippocampus of cKO mice were found (Additional file 1: Fig. S3C, D). These observations indicate that iron-dependent cell death may not be the main cause of hippocampal atrophy in *Hamp*^*GFAP*^ cKO mice.

### Increased microglial number and TNF-α levels but decreased BDNF expression were observed in the hippocampus of *Hamp*^*GFAP*^ cKO mice

Iron accumulation is commonly associated with the activation of inflammation and release of cytokines [29, 30], and cytokines regulate hippocampal neurogenesis [31]. Therefore, we assessed the numbers of astrocytes and microglia in the hippocampal DG. As shown in Additional file 1: Fig. S4, immunofluorescence staining showed a decreased number of GFAP^+^ cells in 3-month-old cKO mice and a decreased number of S100β^+^ mature astrocytes in 14-day-old cKO mice. These findings were consistent with the impairment of NSC development in *Hamp*^*GFAP*^ cKO mice. However, we found that the number of microglia was significantly increased in the hippocampal DG of 3-month-old cKO mice (Fig. 4I, J), as well as in 14-day-old cKO mice (Fig. 4K, L). We determined the mRNA levels of the inflammatory factor interleukin 6 (IL-6) and tumor necrosis factor-α (TNF-α). *IL-6* mRNA levels demonstrated no significant changes, while *TNF-α* mRNA levels increased significantly in the hippocampus of cKO mice (Fig. 4M, N). It was reported that TNF-α plays a significant role in hippocampal development and function, possibly by regulating the expression of neurotrophic factors NGF and BDNF [32]. We therefore evaluated the expression levels of NGF and BDNF. The results showed that both the mRNA and protein levels of BDNF were decreased in the hippocampus of *Hamp*^*GFAP*^ cKO mice (Fig. 4O–Q), while no significant changes were found in the levels of *NGF* mRNA or protein (Additional file 1: Fig. S5).

### Iron overload or *Hamp* knockdown in the hippocampus of WT mice induced TNF-α secretion, BDNF downregulation and neurogenesis impairment

**Fig. 5:**
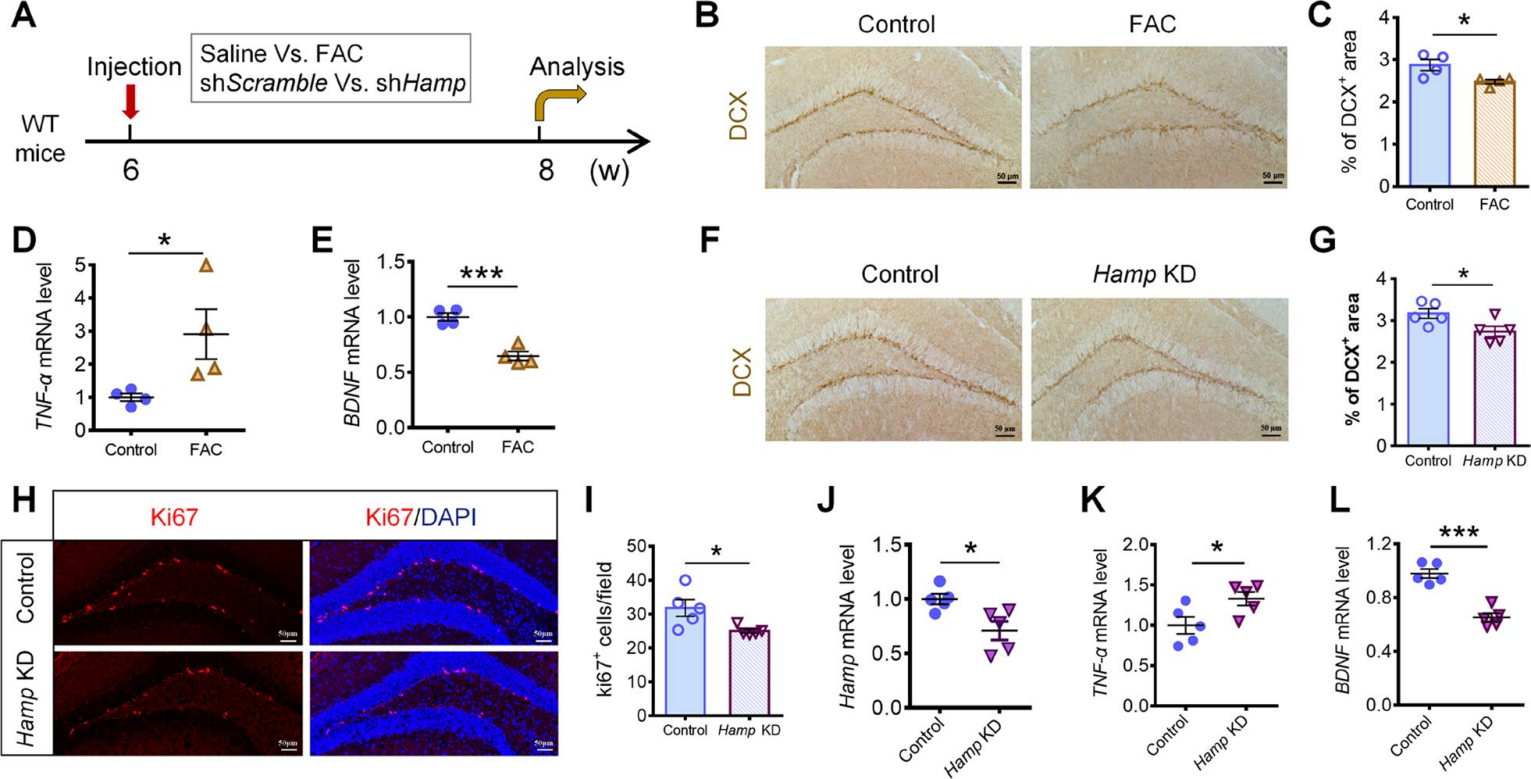
Iron overload or *Hamp* knockdown in the hippocampus of WT mice induced TNF-α secretion and neurogenesis impairment. **A** Schematic diagram showing the injection groups of mice, and time points of treatment and analysis. **B**, **C** Immunohistochemical staining of DCX^+^ cells (**B**) and quantification (**C**) in the hippocampal DG of the control and FAC-treated mice (*n* = 4 per group). **D**, **E**. qRT-PCR detection of the mRNA levels of *TNF-α* (**D**) and *BDNF* (**E**) in the hippocampus of control and FAC-treated mice (*n* = 4 per group). **F**, **G** Immunohistochemical staining of DCX^+^ cells (**F**) and quantification (**G**) in the hippocampal DG of the control and *Hamp* KD mice (*n* = 5 per group). **H**, **I** Immunofluorescence staining images (**H**) and quantification (**I**) of Ki67^+^ cells in the DG region of the control and *Hamp* KD mice (*n* = 5 per group). **J**, **L** qRT-PCR detection of the mRNA levels of *Hamp* (**J**), *TNF-α* (**K**), and *BDNF* (**L**) in the hippocampus of the control and *Hamp* KD mice (*n* = 5 per group). Data were expressed as mean ± SEM. *p < 0.05, ***p < 0.001

To confirm whether the elevated iron content in the hippocampus induced the release of TNF-α and in turn downregulated the expression of BDNF, we established a hippocampal iron overload mouse model by injecting FAC into the hippocampus of 6-week-old C57BL/6 mice, as described previously [20]. The mice were killed 2 weeks after injection for analysis (Fig. 5A). We found that the number of DCX^+^ newborn neurons in the SGZ of FAC-treated mice was significantly lower than that of control mice (Fig. 5B, C), indicating that hippocampal neurogenesis was affected by iron overload. By qRT-PCR, we found that the level of *TNF-α* mRNA increased substantially in the hippocampus of FAC-treated mice, while the level of *BDNF* mRNA decreased significantly (Fig. 5D, E).

Similarly, we knocked down hepcidin expression by injection of pAAV-*GFAP*:sh*Hamp* into the hippocampus of 6-week-old C57BL/6 mice, and analyses were performed 2 weeks later (Fig. 5A). We found that the number of DCX^+^ immature neurons in the SGZ of *Hamp* KD mice was reduced (Fig. 5F, G) and that the number of Ki67^+^ proliferating NSCs was also decreased (Fig. 5H, I). Moreover, after knocking down *Hamp* expression (Fig. 5J), the mRNA level of *TNF-α* increased, while that of *BDNF* decreased significantly in the hippocampus of *Hamp* KD mice (Fig. 5K, L). These findings suggest that iron overload or hepcidin deficiency in the hippocampus can impair hippocampal neurogenesis, possibly by eliciting TNF-α secretion and subsequently downregulating BDNF expression.

### *Nestin*-Cre-mediated* Hamp* knockout mice exhibited pathological changes similar to those shown in *Hamp*^*GFAP*^ cKO mice

**Fig. 6:**
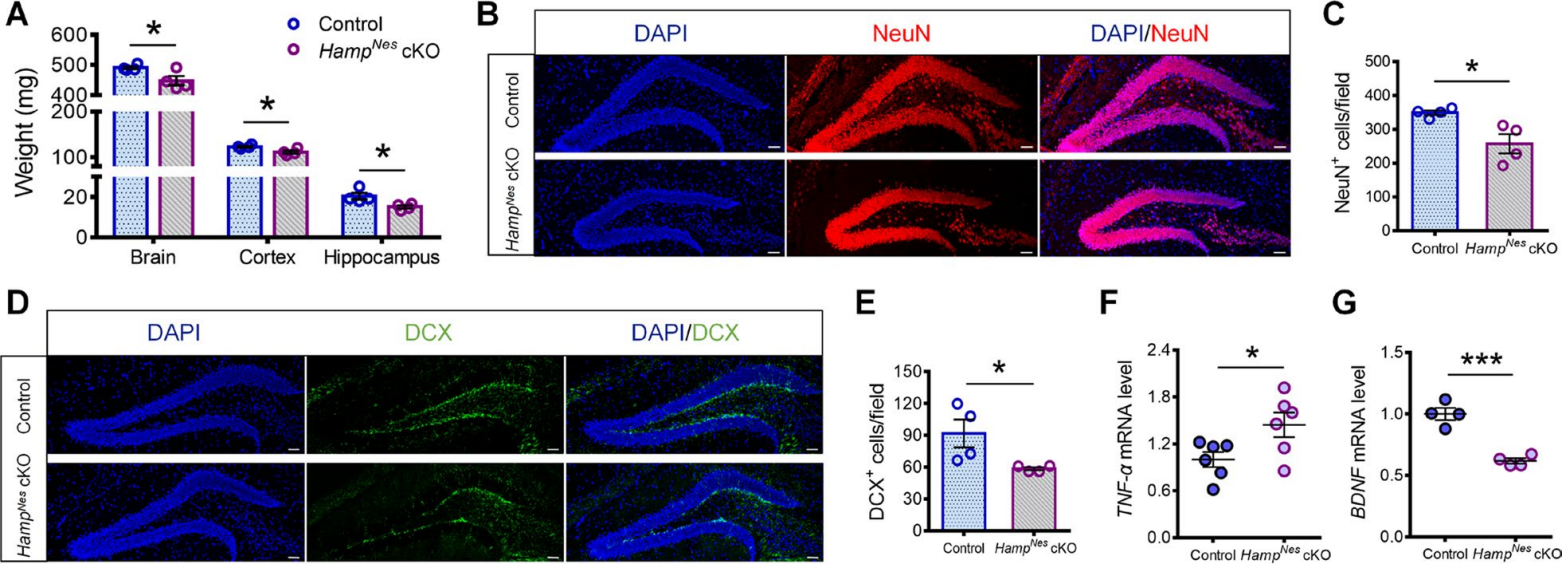
*Nestin*-Cre-mediated *Hamp* knockout mice exhibited pathological changes similar to *Hamp*^*GFAP*^ cKO mice. **A** The weights of total brain, and unilateral cortex and hippocampus of 3-month-old *Hamp*^*Nes*^ cKO mice and control mice. **B**, **C** Immunofluorescence staining of NeuN^+^ cells (**B**) and quantification (**C**) in the hippocampus of *Hamp*^*Nes*^ cKO mice and control mice (scale bar: 50 μm). **D**, **E** Immunofluorescence staining (**D**) and quantification (**E**) of DCX^+^ cells in the DG region of the control and *Hamp*^*Nes*^ cKO mice (scale bar: 50 μm). **F**, **G** qRT-PCR detection of the mRNA levels of *TNF-α* (**F**) and *BDNF* (**G**) in the hippocampus of *Hamp*^*Nes*^ cKO mice and control mice. Data were expressed as mean ± SEM, *n* = 4 or 6 per group. *p < 0.05 and ***p < 0.001

To further confirm the role of hepcidin in hippocampal development, we generated *Nestin*-Cre-mediated *Hamp* cKO mice. The *Hamp*^*Nes*^ cKO mice and control mice were killed at the age of 3 months, and the weights of the whole brain and different brain regions were analyzed. Similar to the *Hamp*^*GFAP*^ cKO mice, the *Hamp*^*Nes*^ cKO mice exhibited brain atrophy, as indicated by the lower weights of the brain, cortex, and hippocampus compared to the control mice (Fig. 6A). Moreover, the number of neurons in the hippocampal DG was significantly reduced (Fig. 6B, C). Impairment of neurogenesis as indicated by a reduction in the number of DCX^+^ cells in the hippocampal SGZ (Fig. 6D, E), accompanied by an increase in *TNF-α* levels (Fig. 6F) and downregulation of *BDNF* (Fig. 6G), was observed in the *Hamp*^*Nes*^ cKO mice. These findings further suggest that hepcidin deficiency in hippocampal NSCs during development can lead to the impairment of neurogenesis.

## Discussion

Iron dysregulation is involved in the pathology of neurological diseases [29, 33]. As the major iron regulator, hepcidin plays an important role in regulating brain iron homeostasis and inflammatory responses [1, 4]. Decreased hepcidin expression has been found in AD patients as well as at the time of disease onset in AD animal models [7]. Increasing the level of hepcidin attenuates oxidative damage in AD models by downregulating brain iron content, and reduces the secretion of TNF-α and IL-6 by astrocytes and microglia [9, 18, 34, 35]. However, whether the decrease in hepcidin levels is a crucial factor contributing to cognitive dysfunction in AD has not been fully explored.

In this study, we generated *Hamp* conditional knockout mice, in which hepcidin was depleted specifically in *GFAP*-Cre-positive cells beginning in the embryonic stage. Therefore, hepcidin was depleted not only in astrocytes, but also in *GFAP*-positive NSCs and dentate granule neurons derived from these cells in the hippocampal DG. Thus, hepcidin expression was substantially reduced in the hippocampus. We found that *Hamp*^*GFAP*^ cKO mice developed severe cognitive impairment, brain atrophy, and a substantial reduction in the number of neuronal cells, particularly in the hippocampal DG. This indicates that a decrease in hepcidin levels beginning in the embryonic stage can severely affect hippocampal development and directly lead to cognitive dysfunction in mice.

The hippocampus is one of the most important brain regions for neurogenesis beginning in the embryonic stage and persisting into adulthood in mammals. It provides substantial structural and functional plasticity to neural circuits and contributes significantly to the regulation of cognition and emotion [22, 36–38]. Increased neurogenesis correlates with improved learning and memory [39, 40], while impaired neurogenesis affects spatial and object recognition and fear conditioning [38, 41]. During hippocampal development, GFAP-positive glial-like NSCs are abundant from embryonic day 13.5 to the early stage of the postnatal period, and furthermore, a lower number of NSCs persist into adulthood, mainly located beneath the granule cells in the DG [22]. In the present study, we found that hippocampal atrophy in *Hamp*^*GFAP*^ cKO mice may be closely associated with impairment of hippocampal neurogenesis. We observed that the numbers of Pax6-positive progenitor cells, Ki67-positive proliferating cells, and DCX-positive immature neurons in the hippocampus of *Hamp*^*GFAP*^ cKO mice were significantly decreased at both embryonic day 18.5 and 3 months. Therefore, decreased proliferation of NSCs and a reduced number of newborn neurons may contribute to structural changes in the hippocampus of *Hamp*^*GFAP*^ cKO mice and thus be associated with cognitive dysfunction and anxiety-like behavior.

Hepcidin has been found to play a dual role in brain iron load and inflammation [42], which suggests that the inflammation status may determine whether hepcidin has deleterious or beneficial effects in the brain. Hepcidin plays a protective role in normal and progressive iron overload models, such as AD [8–10, 17, 34], while it exerts a detrimental effect in acute iron overload and inflammation models [12, 43, 44]. To clarify the changes in iron metabolism in the brains of *Hamp*^*GFAP*^ cKO mice, iron content and distribution were assessed. ICP-MS showed an increased iron level in the hippocampus of cKO mice, while Perl’s stain showed that iron levels in the hippocampal DG were notably increased. Furthermore, the increased expression of the iron storage protein FtL was consistent with the increase in iron levels in the hippocampus of *Hamp*^*GFAP*^ cKO mice [45]. The increase of brain iron content in *Hamp*^*GFAP*^ cKO mice was consistent with that observed in *Hamp*^*−/−*^ mice in our previous study [17]. These findings suggest that hepcidin knockout in astrocytes in *Hamp*^*GFAP*^ cKO mice may facilitate iron uptake at the BBB, resulting in more iron entry into brain tissue [17]. Interestingly, the weight of the cortex did not show significant differences between the *Hamp*^*GFAP*^ cKO mice and the control mice, indicating that iron dysregulation in the brains of cKO mice had a greater impact on hippocampal development.

We then investigated how the elevated brain iron level led to the impairment of hippocampal neurogenesis. Iron overload is often associated with the activation of apoptosis and increased inflammation [26, 29, 30]. However, in the hippocampus of *Hamp*^*GFAP*^ cKO mice, we did not observe a significant increase in the number of apoptotic cells, activation of caspase-3, or levels of ferroptosis-related proteins. These findings indicate that a decrease in the number of proliferating NSCs, rather than iron-induced cell death, is the main cause of impaired neurogenesis in *Hamp*^*GFAP*^ cKO mice. Furthermore, we found that the inflammatory response was increased in the hippocampus of *Hamp*^*GFAP*^ cKO mice, as evidenced by increases in the activation of microglia and TNF-α secretion. This is consistent with the anti-inflammatory effects of hepcidin in progressive iron overload disease models [8–10, 34].

Hippocampal neurogenesis is tightly regulated by intrinsic signaling pathways and extrinsic environmental factors, including morphogens, growth factors, neurotrophins, cytokines, and hormones [46–48]. The secretion of proinflammatory cytokines, such as IL-6, IL-1β, IL-12, and TNF-ɑ, exerts anti-neurogenic effects by decreasing NSC proliferation [49–51]. In the hippocampus of *Hamp*^*GFAP*^ cKO mice, in addition to the number of microglia being increased, TNF-α levels increased significantly. Studies have shown that TNF-α is involved in the development and function of the hippocampus [52, 53]. TNF-α can specifically inhibit hippocampal neurogenesis [54–56], while reduced concentrations of TNF-α promote neurogenesis by regulating the expression of growth factors and neurotrophins [32, 57, 58]. Therefore, the increase in TNF-α levels induced by *GFAP*-Cre-mediated hepcidin depletion may account for the decrease in the proliferation of hippocampal NSCs, thus contributing to abnormal hippocampal development and cognitive decline in mice.

The pathological changes in the *Hamp*^*GFAP*^ cKO mice and the underlying mechanism were further tested by using a hippocampal iron overload mouse model and *Hamp* KD mice. In both cases, hippocampal neurogenesis was affected, as indicated by a reduced number of DCX-positive cells compared to the respective control mice. Moreover, TNF-α secretion increased and BDNF levels decreased. These findings confirmed the role of hepcidin deficiency and the resulting iron overload in the impairment of hippocampal neurogenesis, as well as the involvement of TNF-α upregulation and BDNF downregulation in the underlying molecular mechanism. Furthermore, these changes were also observed in *Nestin*-Cre-mediated *Hamp* cKO mice, indicating that impairment of hippocampal structure and function was mainly due to hepcidin deficiency-induced abnormal hippocampal neurogenesis.

## Conclusions

The present study showed that hepcidin depletion in *GFAP*-positive cells during development induced hippocampal atrophy and cognitive decline in mice. The molecular mechanism might involve an increase in brain iron content due to increased iron uptake at the BBB after hepcidin knockout. Iron accumulation activated microglia and promoted the secretion of TNF-α, which in turn downregulated BDNF and impaired hippocampal neurogenesis, leading to a substantial decrease in the number of neurons and eventually impairing the function of the hippocampus. This study provides an important potential mechanism for the negative impact of hepcidin deficiency on neurodevelopment and cognitive function, verifying the role of iron and associated inflammatory cytokines as key modulators of hippocampal neurogenesis.

### Supplementary Information

**Additional file 1: Table S1.** Primer sequences for genotyping. **Table S2.** Primer sequences for qRT-PCR. **Figure S1.** Genotyping and behavioral tests of *Hamp*^*GFAP*^ cKO and control mice. **A** PCR amplification for identifying the bands of *Hamp*^*flox/flox*^ (homozygote: one band at 201 bp; heterozygote: two bands at 201 bp and 142 bp; wildtype allele: one band at 142 bp) and GFAP-Cre (with cre: 700 bp; wildtype: no band). **B**–**E** In the Morris water maze test, the escape latency (**B**) and distance (**C**) during the training stage, and the percentage of time spent in the target quadrant (**D**) and number of times crossing the platform (**E**) of the 6-month-old *Hamp*^*GFAP*^ cKO and control mice were analyzed. **F**–**I **In the elevated plus maze test, the percentages of time (**F**) and distance (**G**) spent in the open zone, and the percentages of time (**H**) and distance (**I**) in the center area of the 3-month-old *Hamp*^*GFAP*^ cKO and control mice were analyzed. Data were expressed as mean ± SEM, *n* = 6 per group. *p < 0.05 and **p < 0.01. **Figure S2.** Body weights and the Nissl staining results of the *HampGFAP *cKO and control mice. **A–C** The body weights of 3-month-old (**A**, *n* = 6), 6-month-old (**B**, *n* = 7), and 14-day-old (**C**, *n* = 7 and 5) *Hamp*^*GFAP*^ cKO and control mice were determined. **D**, **E **Representative Nissl staining images (**D**) and quantification (**E**) in the 3-month-old *Hamp*^*GFAP*^ cKO and control mice (*n* = 3). Data were expressed as mean ± SEM. ***p < 0.001. **Figure S3.** Detection of apoptosis and ferroptosis levels in the hippocampus of *Hamp*^*GFAP*^ cKO and control mice. **A**, **B** TUNEL staining (**A**) and cleaved Caspase-3 immunostaining (**B**) for detecting apoptotic cells in the hippocampal DG of 3-month-old *Hamp*^*GFAP*^ cKO and control mice (scale bar: 50 μm). **C**, **D** Western blot images (**C**) and quantification (**D**) of GPx4 and ACSL4 expression levels in the hippocampus of 3-month-old *Hamp*^*GFAP*^ cKO and control mice (*n* = 4 per group, GAPDH and β-actin as the internal reference). Data were expressed as mean ± SEM. **Figure S4.** Immunostaining for astrocytes in the hippocampus of *Hamp*^*GFAP*^ cKO and control mice. **A**, **B** Representative immunostaining images for GFAP (**A**) and quantification (**B**) in the hippocampal DG of 3-month-old *Hamp*^*GFAP*^ cKO and control mice (scale bar: 50 μm, *n* = 4 per group). **C**, **D **Representative immunostaining images for S100β (**C**) and quantification (**D**) in the hippocampal DG of 14-day-old *Hamp*^*GFAP*^ cKO and control mice (scale bar: 50 μm, *n* = 5 per group). Data were expressed as mean ± SEM. *p < 0.05. **Figure S5**. Detection of *NGF* mRNA and protein levels in the hippocampus of *Hamp*^*GFAP*^ cKO and control mice. **A** qRT-PCR results of NGF expression in the hippocampus of 3-month-old *Hamp*^*GFAP*^ cKO and control mice (*n* = 4 per group). **B**, **C** Western blot images (**B**) and quantification (**C**) of NGF expression levels in the hippocampus of 3-month-old *Hamp*^*GFAP*^ cKO and control mice (*n* = 4 per group; β-actin as the internal reference). Data were expressed as mean ± SEM.

## Data Availability

The data that support the findings of this study are included in this published article and its Additional file. Other materials are available from the corresponding authors on reasonable request.

## Abbreviations

FPN1: Ferroportin 1
AD: Alzheimer’s disease
BBB: Blood–brain barrier
NSCs: Neural stem/progenitor cells
SGZ: Subgranular zone
DG: Dentate gyrus
WT: Wild type
FAC: Ferric ammonium citrate
MWM: Morris water maze
DCX: Doublecortin
FtL: Ferritin light chain
TUNEL: Terminal deoxynucleotidyl transferase-mediated dUTP nick-end labeling
GPx4: Glutathione peroxidase 4
ACSL4: Acyl-CoA synthetase long-chain family member 4
NGF: Nerve growth factor
BDNF: Brain-derived neurotrophic factor
qR-PCR: Quantitative reverse-transcription PCR
ICP-MS: Inductively coupled plasma-mass spectrometry
IL-6: Interleukin 6
TNF-α: Tumor necrosis factor-α
