# Supplementary material for: Hepcidin deficiency impairs hippocampal neurogenesis and mediates brain atrophy and memory decline in mice
Source: J Neuroinflammation. 2024 Jan 9;21:15. doi: 10.1186/s12974-023-03008-0 (PMC10777572; doi:10.1186/s12974-023-03008-0)
Supplement: Supplementary file 1 — Additional file 1: Table S1. Primer sequences for genotyping. Table S2. Primer sequences for qRT-PCR. Figure S1. Genotyping and behavioral tests of HampGFAP cKO and control mice. A PCR amplification for identifying the bands of Hampflox/flox (homozygote: one band at 201 bp; heterozygote: two bands at 201 bp and 142 bp; wildtype allele: one band at 142 bp) and GFAP-Cre (with cre: 700 bp; wildtype: no band). B–E In the Morris water maze test, the escape latency (B) and distance (C) during the training stage, and the percentage of time spent in the target quadrant (D) and number of times crossing the platform (E) of the 6-month-old HampGFAP cKO and control mice were analyzed. F–I In the elevated plus maze test, the percentages of time (F) and distance (G) spent in the open zone, and the percentages of time (H) and distance (I) in the center area of the 3-month-old HampGFAP cKO and control mice were analyzed. Data were expressed as mean ± SEM, n = 6 per group. *p < 0.05 and **p < 0.01. Figure S2. Body weights and the Nissl staining results of the HampGFAP cKO and control mice. A–C The body weights of 3-month-old (A, n = 6), 6-month-old (B, n = 7), and 14-day-old (C, n = 7 and 5) HampGFAP cKO and control mice were determined. D, E Representative Nissl staining images (D) and quantification (E) in the 3-month-old HampGFAP cKO and control mice (n = 3). Data were expressed as mean ± SEM. ***p < 0.001. Figure S3. Detection of apoptosis and ferroptosis levels in the hippocampus of HampGFAP cKO and control mice. A, B TUNEL staining (A) and cleaved Caspase-3 immunostaining (B) for detecting apoptotic cells in the hippocampal DG of 3-month-old HampGFAP cKO and control mice (scale bar: 50 μm). C, D Western blot images (C) and quantification (D) of GPx4 and ACSL4 expression levels in the hippocampus of 3-month-old HampGFAP cKO and control mice (n = 4 per group, GAPDH and β-actin as the internal reference). Data were expressed as mean ± SEM. Figure S4. Immunostaining for astrocytes [file 12974_2023_3008_MOESM1_ESM.pdf]

Supplementary tables and figures

Table S1. Primer sequences for genotyping.

Primers	Sequences (5′-3′)
Hamp^{flox} forward	GTAGGGTCTGATAAGTGAAGCCAG
Hamp^{flox} reverse	AGCACAAAGGCTTATAGCACATTC
GFAP-Cre forward	TAGCCCACTCCTTCATAAAGCCCT
GFAP-Cre reverse	GCTAAGTGCCTTCTCTACACC
Nestin-Cre forword	CCTTCCTGAAGCAGTAGAGCA
Nestin-Cre reverse	GCCTTATTGTGGAAGGACTG

Table S2. Primer sequences for qRT-PCR.

Genes	Forward (5′-3′)	Reverse (5′-3′)
GAPDH	TGACTTCAACAGCGACACCCA	CACCCTGTTGCTGTAGCCAAA
Hamp	CTGAGCAGCACCACCTATCTC	TGGCTCTAGGCTATGTTTTGC
TNF-α	CACCATGAGCACAGAAAGCA	TAGACAGAAGAGCGTGGTGG
IL-6	CTGCAAGAGACTTCCATCCAG	AGTGGTATAGACAGGTCTGTTGG
BDNF	CTGTATCAAAAGGCCAACTGAA	GTGTCTATCCTTATGAATCGCCA
NGF	ACTGGACTAAACTTCAGCATTCC	GGGCAGCTATT GGTGCAGTA

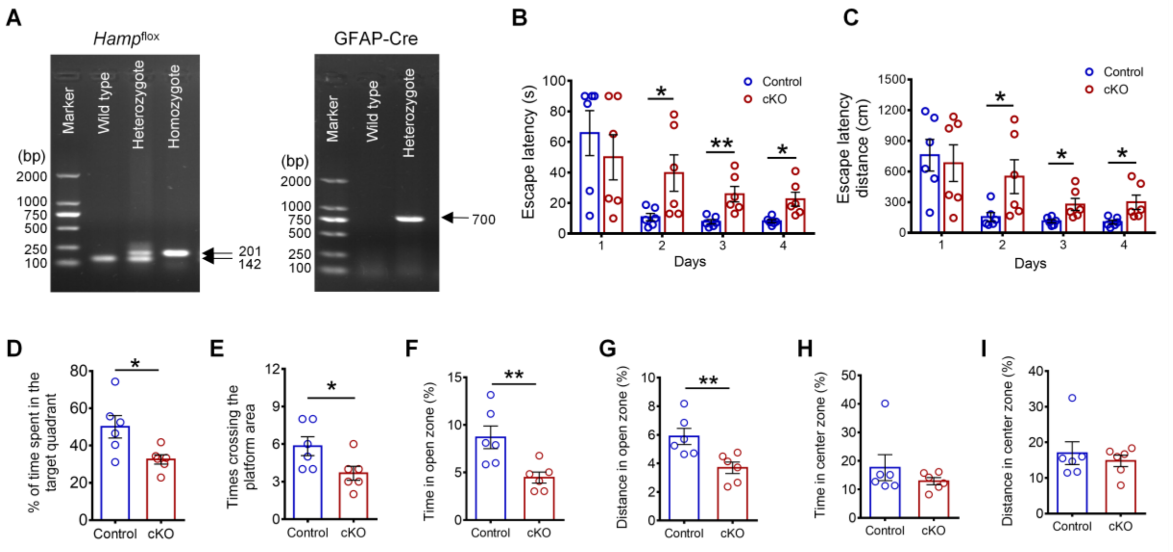

Figure S1. Genotyping and behavioral tests of *Hamp*^{GFAP} cKO and control mice. A PCR amplification for identifying the bands of *Hamp*$^{flox/flox}$ (homozygote: one band at 201 bp; heterozygote: two bands at 201 bp and 142 bp; wildtype allele: one band at 142 bp) and GFAP-Cre (with cre: 700 bp; wildtype: no band). **B-E** In the Morris water maze test, the escape latency (B) and distance (C) during the training stage, and the percentage of time spent in the target quadrant (D) and number of times crossing the platform (E) of the 6-month-old *Hamp*^{GFAP} cKO and control mice were analyzed. **F-I** In the elevated plus maze test, the percentages of time (F) and distance (G) spent in the open zone, and the percentages of time (H) and distance (I) in the center area of the 3-month-old *Hamp*^{GFAP} cKO and control mice were analyzed. Data were expressed as mean ± SEM, n = 6 per group. *$p < 0.05$ and **$p < 0.01$.

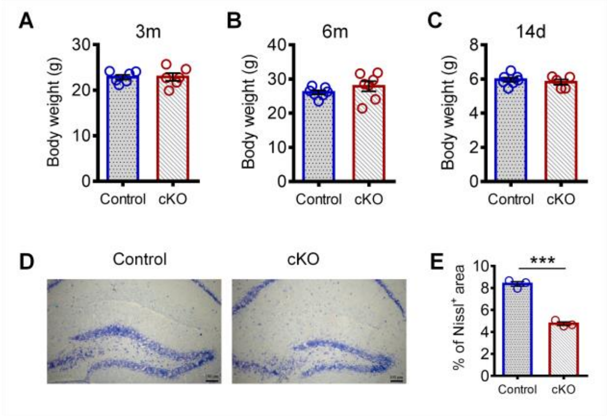

Figure S2. Body weights and the Nissl staining results of the *Hamp*^{GFAP} cKO and control mice. A-C The body weights of 3-month-old (A, n = 6), 6-month-old (B, n = 7), and 14-day-old (C, n = 7 and 5) *Hamp*^{GFAP} cKO and control mice were determined. **D-E** Representative Nissl staining images (D) and quantification (E) in the 3-month-old *Hamp*^{GFAP} cKO and control mice (n = 3). Data were expressed as mean ± SEM. ***$p < 0.001$.

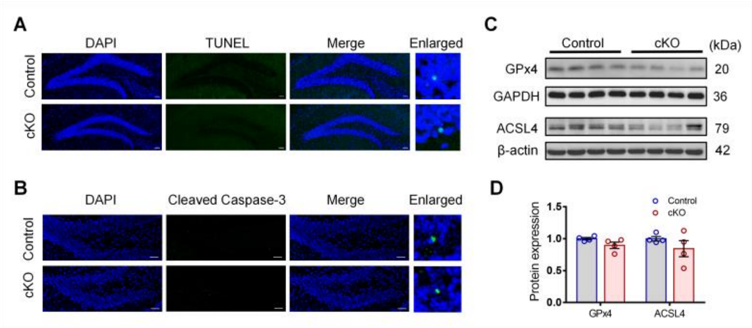

Figure S3. Detection of apoptosis and ferroptosis levels in the hippocampus of *Hamp*^{GFAP} cKO and control mice. A-B TUNEL staining (A) and cleaved Caspase-3 immunostaining (B) for detecting apoptotic cells in the hippocampal DG of 3-month-old *Hamp*^{GFAP} cKO and control mice (scale bar: 50 μm). **C-D** Western blot images (C) and quantification (D) of GPx4 and ACSL4 expression levels in the hippocampus of 3-month-old *Hamp*^{GFAP} cKO and control mice (n = 4 per group, GAPDH and β-actin as the internal reference). Data were expressed as mean ± SEM.

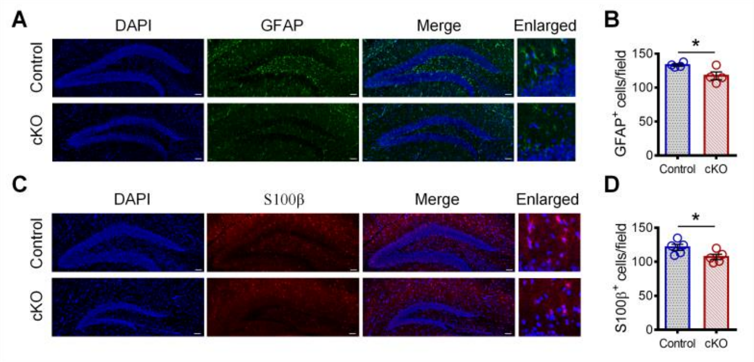

Figure S4. Immunostaining for astrocytes in the hippocampus of *Hamp*^{GFAP} cKO and control mice. A-B Representative immunostaining images for GFAP (A) and quantification (B) in the hippocampal DG of 3-month-old *Hamp*^{GFAP} cKO and control mice (scale bar: 50 μm, n = 4 per group). **C-D** Representative immunostaining images for S100β (C) and quantification (D) in the hippocampal DG of 14-day-old *Hamp*^{GFAP} cKO and control mice (scale bar: 50 μm, n = 5 per group). Data were expressed as mean ± SEM. *$p < 0.05$.

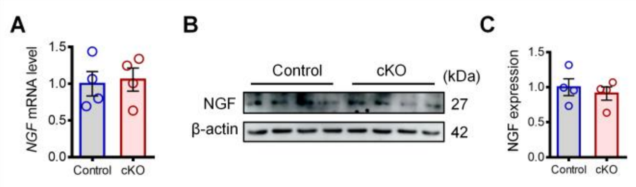

Figure S5. Detection of *NGF* mRNA and protein levels in the hippocampus of *Hamp*^{GFAP} cKO and control mice. A qRT-PCR results of *NGF* expression in the hippocampus of 3-month-old *Hamp*^{GFAP} cKO and control mice (n = 4 per group). **B-C** Western blot images (B) and quantification (C) of NGF expression levels in the hippocampus of 3-month-old *Hamp*^{GFAP} cKO and control mice (n = 4 per group; β-actin as the internal reference). Data were expressed as mean ± SEM.
